# Structural analyses of 2015-updated drug-resistant mutations in HIV-1 protease: an implication of protease inhibitor cross-resistance

**DOI:** 10.1186/s12859-016-1372-3

**Published:** 2016-12-22

**Authors:** Chinh Tran-To Su, Wei-Li Ling, Wai-Heng Lua, Yu-Xuan Haw, Samuel Ken-En Gan

**Affiliations:** 1 Bioinformatics Institute, Agency for Science, Technology, and Research (A*STAR), Singapore, 138671 Singapore; 2 p53 Laboratory, Agency for Science, Technology, and Research (A*STAR), Singapore, 138648 Singapore

## Abstract

**Background:**

Strategies to control HIV for improving the quality of patient lives have been aided by the Highly Active Anti-Retroviral Therapy (HAART), which consists of a cocktail of inhibitors targeting key viral enzymes. Numerous new drugs have been developed over the past few decades but viral resistances to these drugs in the targeted viral enzymes are increasingly reported. Nonetheless the acquired mutations often reduce viral fitness and infectivity. Viral compensatory secondary-line mutations mitigate this loss of fitness, equipping the virus with a broad spectrum of resistance against these drugs. While structural understanding of the viral protease and its drug resistance mutations have been well established, the interconnectivity and development of structural cross-resistance remain unclear. This paper reports the structural analyses of recent clinical mutations on the drug cross-resistance effects from various protease and protease inhibitors (PIs) complexes.

**Methods:**

Using the 2015 updated clinical HIV protease mutations, we constructed a structure-based correlation network and a minimum-spanning tree (MST) based on the following features: (i) topology of the PI-binding pocket, (ii) allosteric effects of the mutations, and (iii) protease structural stability.

**Results and conclusion:**

Analyis of the network and the MST of dominant mutations conferring resistance to the seven PIs (Atazanavir-ATV, Darunavir-DRV, Indinavir-IDV, Lopinavir-LPV, Nelfinavir-NFV, Saquinavir-SQV, and Tipranavir-TPV) showed that cross-resistance can develop easily across NFV, SQV, LPV, IDV, and DRV, but not for ATV or TPV. Through estimation of the changes in vibrational entropies caused by each reported mutation, some secondary mutations were found to destabilize protease structure. Our findings provide an insight into the mechanism of PI cross-resistance and may also be useful in guiding the selection of PI in clinical treatment to delay the onset of cross drug resistance.

**Electronic supplementary material:**

The online version of this article (doi:10.1186/s12859-016-1372-3) contains supplementary material, which is available to authorized users.

## Background

Ever since the identification of the Human Immunodeficiency Virus (HIV) as the cause of Acquired Immunodeficiency Syndrome (AIDS), scientists have raced to find effective and sustainable treatment methods to inhibit viral replication and assembly. In the last 30 years alone, HIV has grown to be a pandemic with more than 35 million people infected worldwide [1]. While therapeutic progress has been made in prolonging the lifespan of infected individuals using the Highly Active Anti-Retroviral Therapy (HAART) [2–4], HIV rapidly adapts and develops drug resistance.

Although new drugs such as Protease Inhibitors (PIs) are constantly being developed, such progress is outpaced by HIV drug resistance. This drug resistance arises from mutations in the viral protease gene to compromise the protease-PI interaction to facilitate the binding to protease substrate (i.e. Gag), even in the presence of the PIs [5–11]; consequently rendering the PIs ineffective.

While drug resistant mutations are often associated to specific PIs, many confer cross-resistance to other PIs [12]. The cross-resistance makes it challenging to map specific protease mutations to specific PIs. Nevertheless, to effectively guide clinical selection of second or third line of treatment when resistance to one such PI has occurred, such investigations are necessary [13]. Mutation mappings have revealed that these mutations spontaneously arise as part of the natural variance [14] and become dominant during PI-drug treatments.

This high variance in the HIV enzymes often results in reduced viral fitness (in terms of replication and infectivity) and an increasing percentage of inactivated or “unfit” viruses [15]. Nonetheless, of the PI-resistance mutations [16], many also compensate for the reduced viral fitness [17–20]. Such compensatory mutations are typically found outside the protease active site or on the protease substrate Gag [21–26] to balance fitness with the impaired enzymatic activity. Through better surveillance, the reports of such emerging mutations would certainly enable in-depth investigations into the structural mechanisms of drug resistance [27, 28].

Current studies of protease structures bearing different resistance mutations [7, 18–20, 27–33] are generally focussed on the protease flaps located above the protease active site and how the flaps mediate PI accessibility. Mutations in this area naturally affect the flap motions, reducing PI accessibility and binding. While such mutations can be single point mutations or a cluster to confer resistances to specific PIs, the exact structural mechanisms that result in cross-drug resistance remain enigmatic.

Using network analyses, Ragland et al. [27] and Appadurai et al. [28] investigated the relationship of mutations inside and outside the protease active site. Their studies revealed allosteric effects that explained resistance development against the current PIs. While their studies conveyed residue-based correlations within a protease structure and for specific PI resistance, the cross-resistance conferred by different combinations of mutations remains not well established.

In this study, we investigated different combinations of protease mutations and how they lead to resistance to various PIs by constructing a structure-based correlation network of 33 protease-PI mutant complexes from the 2015-updated clinical drug-resistant mutations in HIV-1 protease [34]. To construct this network, we employed different structural constraints such as PI-binding pockets corresponding to various PIs, allosteric effects on the PI-binding region, and structural stability affected by mutations. In addition, we investigated the mutation hotspots that can destabilize the protease structure. Together, these findings can lead to better understanding of PI cross-resistance and serve as a possible guide in the clinical selection of PIs to delay the onset of PI resistance.

## Methods

### Structural modelling of mutated complexes of protease and protease inhibitors (PIs)

We modelled 26 mutated protease structures based on the various combinations of mutations reported in the 2015 update of drug-resistance mutations in HIV-1 [34]. According to this report, the mutations were categorized into “major” (substantially reducing protease binding ability to the PIs) and “minor” (some compensate and improve the viral fitness loss caused by major mutations, whereas others affect PI susceptibility) [34]. These protease mutants are known to be resistant to the seven Protease Inhibitors (PIs): Atazanavir (ATV), Darunavir (DRV), Indinavir (IDV), Lopinavir (LPV), Nefinavir (NFV), Saquinavir (SQV), and Tipranavir (TPV).

As starting points for computational mutagenesis, seven structures of protease-PI complexes from the Protein Data Bank (PDB) for the corresponding PIs were selected: ATV [PDB:3OXX], DRV [PDB:4LL3], IDV [PDB:2B7Z], LPV [PDB:2Q5K], NFV [PDB:1OHR], SQV [PDB:3S56], and TPV [PDB:3SPK]. For each protease-PI complex, we used SCWRL4 [35] to model the side chains of the mutated residues in the protease. The PI coordinates were then input into the modelled protease and minimized using AMBER12 [36].

### Constructing the correlation network of the modelled protease-PI complexes

We simulated the co-related drug resistant mutations against various PIs by constructing a weighted graph generated by Gephi v0.82 [37]. The graph *G = (V, E)* contains a set of nodes *V*, each of which represents a protease-PI complex containing the combinations of major and/or minor protease mutations [34]. Nodes are connected by edges *E* represented by the pairwise structural co-relationships between nodes.

To investigate the influence among the nodes, we assigned node weights using *closeness centrality*, which quantifies the closeness of a node to the other nodes in the network. The nodes with high *closeness centrality* show the potentially related mutations that would be selected in resistance development against various PIs.

For each node *v* in graph *G*, we characterized a vector *C*_*v*_ involving (i) PI-binding pocket corresponding to various PIs and mutations, (ii) allosteric effects on the PI-binding region caused by the mutations, and (iii) structural stability affected by the mutations.

(i) PI-binding pocket
  The protein cavity detection package *fpocket* [38] was used to estimate the PI-binding pocket volume (*Vpocket*) for each protease-PI variant and the influence of the different mutation categories (i.e. major or minor) on the PI-binding region.
(ii) Allosteric effect
  SPACER [39] was used to detect communications and the impact on the PI-binding regions resulting from the major and minor mutations. The communication strengths between sites, characterized by leverage coupling (details in [40]), imply the potential allosteric effect (*AllosComm* defined in our *C*_*v*_ vector) caused by the various mutations.
  Due to the different combinations of mutations, where some minor mutations do affect the major mutations and PI susceptibility, we estimated the *AllosComm* as below:
  1. **If** the protease-PI complex contains *major* mutations **do**
  2.  *AllosComm* ← effect of both (*major, minor*) to the PI-binding region, excluding effect of *minor* onto the *major* mutations
  3. **Else:**
  4.  *AllosComm* ← effect of *minor* to the PI-binding region
  5. **End if**
(iii) Structural stability
  Thermostability of a native protease [PDB:1ODW] given the various mutations was evaluated using ENCoM server [41] and the resulting free energy (*ΔΔG*) was assigned to the mutated protease structure by linearly combining all energy values (including vibrational entropy and approximated enthalpy scores) that resulted from each single mutation.
  The structural deviations (*RMSd*) of the resulting mutated protease from the native protease above were also calculated.
  Therefore in our graph *G,* we defined an integrated vector *C*_*v*_ for each node *v* as
  C_v_ = (*Vpocket, AllosComm, ΔΔG, RMSd*), and the weight of the edge between nodes *i,j* was estimated based on Pearson’s correlation between two normalized vectors *C*_*vi*_ and *C*_*vj*_ *.*

### Constructing the minimum spanning tree

After constructing the graph *G*, a shortest path subgraph *H* was extracted by employing the Kruskal’s minimum spanning tree (MST) algorithm [42] in Scipy package [43]. In this subgraph *H*, nodes were weighted using numbers of neighbours, and the edges were defined the same as in *G*.

## Results and discussion

### Structural relationship of PI-resistance protease mutants

We set out to study the cross-drug resistance of protease mutations associated with various PIs structurally. To do this, we estimated the protease pocket volumes of 33 protease-PI complexes using *fpocket* [38] and calculated the pocket volume for another seven native protease-PI complexes [referenced from PDB:1ODW]. Results of the seven native complexes showed that the protease pocket volume was capable of flexible adjustment in accordance to the PI size (Tables 1, 2, 3 and 4) and that even a single mutation can alter the pocket volume of HIV protease (see Additional file 1).

**Table 1:** Structural characterized features used in the graph constructions for the PI-resistance mutants to ATV and DRV

	Vpocket^a^ (Å^3^)	AllosComm^b^	ΔΔG score / ΔS^c^ (kcal/mol)	RMSd (Å)
Atazanavir (ATV) — molecular weight = ~705 g/mol				
ATV_0 (major/minor)	**2484.44** (2325.65)	0.721 (0.402)	1.13 / −0.13	0.93
ATV_1 (major/minor)	1675.04	0.848 (0.761)	8.74 / −3.20	1.11
ATV_2 (minor)	1779.57	0.206 (0.668)	5.21 / −1.75	1.07
ATV_3 (minor)	2009.24	0.268 (0.81)	1.94 / −1.93	1.05
ATV_4 (minor)	1895.13	0.206 (0.756)	1.50 / 0.02	1.06
ATV_5 (minor)	1884.48	0.014 (0.879)	0.30 / −0.009	1.01
Darunavir (DRV) — molecular weight = ~548 g/mol				
DRV_0 (wt)	1310.9 (1842.30)	0.692 (0.269)	−1.31 / −0.59	0.83
DRV_1 (major/minor)	1570.52	0.667 (0.635)	3.54 / 1.91	1.09
DRV_2 (major)	1636.82	0.521 (0.765)	−0.13 / −0.06	1.07

^a^Pocket volume of the native protease [PDB: 1ODW], which contains no mutations in the presence of corresponding PIs, is shown in parentheses for comparison purpose. In the cases of wild type proteases DRV_0, there is a single mutation found (i.e. S37N) in the structures when compared to the native protease sequence. The complexes that contain the quadruple mutants and show increased pocket volume are in bold

^b^For comparison purposes of allosteric effect, communication estimated values of the native protease were shown in parentheses

^c^ΔΔG and ΔS scores represent free energy and vibrational energy respectively, demonstrating thermo-stability of the protease when mutated from the native protease

**Table 2:** Structural characterized features used in the graph constructions for the PI-resistance mutants to IDV and LPV

	Vpocket^a^ (Å^3^)	AllosComm^b^	ΔΔG score / ΔS^c^ (kcal/mol)	RMSd (Å)
Indinavir (IDV) — molecular weight = ~614 g/mol				
IDV_0 (major/minor)	**1929.67** (1863.57)	0.989 (0.745)	−1.37 / −1.18	1.14
IDV_1 (major/minor)	**1884.57**	0.770 (0.825)	4.32 / −0.45	1.09
IDV_2 (major/minor)	1769.62	0.518 (0.468)	2.14 / 0.72	1.04
Lopinavir (LPV) — molecular weight = ~629 g/mol				
LPV_0 (wt)	1659.68 (2201.32)	0.696 (0.257)	0.13 / 0.34	0.88
LPV_1 (major/minor)	**1872.66**	0.590 (0.547)	4.53 / 0.58	1.0
LPV_2 (major/minor)	1867.24	0.665 (0.452)	1.51 / −0.15	0.98
LPV_3 (major/minor)	1781.42	0.8 (0.646)	4.28 / 1.47	1.06
LPV_4 (major/minor)	2029.70	0.769 (0.646)	1.51 / 0.48	1.08
LPV_5 (minor)	2051.45	0.167 (0.944)	1.28 / 0.62	1.08
LPV_6 (minor)	2034.89	0.385 (0.944)	2.27 / 0.78	1.08

^a^Pocket volume of the native protease [PDB: 1ODW], which contains no mutations in the presence of corresponding PIs, is shown in parentheses for comparison purpose. In the cases of wild type proteases LPV_0, there is a single mutation found (i.e. L63P) in the structures when compared to the native protease sequence. The complexes that contain the quadruple mutants and show increased pocket volume are in bold

^b^For comparison purposes of allosteric effect, communication estimated values of the native protease were shown in parentheses

^c^ΔΔG and ΔS scores represent free energy and vibrational energy respectively, demonstrating thermo-stability of the protease when mutated from the native protease

**Table 3:** Structural characterized features used in the graph constructions for the PI-resistance mutants to NFV and SQV

	Vpocket^a^ (Å^3^)	AllosComm^b^	ΔΔG score / ΔS^c^ (kcal/mol)	RMSd (Å)
Nelfinavir (NFV) — molecular weight = ~568 g/mol				
NFV_0 (wt)	1637.23 (1823.01)	0.584 (0.101)	−1.23 / −0.58	0.6
NFV_1 (major/minor)	1841.98	0.655 (0.403)	2.08 / −0.70	1.09
NFV_2 (minor)	1634.29	0.308 (0.312)	0.62 / −0.63	1.03
NFV_3 (minor)	1669.08	0.795 (0.713)	0.51 / 0.28	0.98
NFV_4 (minor)	1692.91	0.851 (0.713)	0.68 / 0.21	0.98
Saquinavir (SQV) — molecular weight = ~671 g/mol				
SQV_0 (minor)	1735.73 (2193.99)	0.735 (0.38)	3.27 / 1.46	0.84
SQV_1 (major/minor)	1700.44	0.787 (0.371)	6.02 / −1.89	0.81
SQV_2 (minor)	1631.92	0.601 (0.346)	0.22 / −0.61	0.82
SQV_3 (minor)	1817.81	0.739 (0.441)	2.14 / 0.72	0.79
SQV_4 (minor)	1531.07	0.748 (0.606)	0.67 / 0.21	0.8

^a^Pocket volume of the native protease [PDB: 1ODW], which contains no mutations in the presence of corresponding PIs, is shown in parentheses for comparison purpose. In the cases of wild type proteases NFV_0, there is a single mutation found (i.e. V3I) in the structures when compared to the native protease sequence. The complexes that contain the quadruple mutants and show increased pocket volume are in bold

^b^For comparison purposes of allosteric effect, communication estimated values of the native protease were shown in parentheses

^c^ΔΔG and ΔS scores represent free energy and vibrational energy respectively, demonstrating thermo-stability of the protease when mutated from the native protease

**Table 4:** Structural characterized features used in the graph constructions for the PI-resistance mutants to TPV

	Vpocket^a^ (Å^3^)	AllosComm^b^	ΔΔG score / ΔS^c^ (kcal/mol)	RMSd (Å)
Tipranavir (TPV) — molecular weight = ~603 g/mol				
TPV_0 (major/minor)	1912.49 (1963.76)	0.627 (0.277)	3.54 / 0.42	0.97
TPV_1 (major/minor)	1858.07	0.741 (0.795)	8.99 / 3.12	1.03
TPV_2 (major/minor)	1804.05	0.905 (0.287)	0.90 / 0.7	1.05
TPV_3 (minor)	2228.93	0.24 (0.379)	4.43 / 1.3	1.02

^a^Pocket volume of the native protease [PDB: 1ODW], which contains no mutations in the presence of corresponding PIs, is shown in parentheses for comparison purpose

^b^For comparison purposes of allosteric effect, communication estimated values of the native protease were shown in parentheses

^c^ΔΔG and ΔS scores represent free energy and vibrational energy respectively, demonstrating thermo-stability of the protease when mutated from the native protease

In the three wild type protease-PI complexes: DRV_0, LPV_0, and NFV_0, naturally occurring single residue substitutions S37N, L63P, and V3I, respectively, were found to shrink the PI-binding pocket (~30% decrease in DRV_0 and LPV_0, and ~10% decrease in NFV_0). This may explain the strong binding of wild-type proteases with the PIs (with low dissociation constant K_d_ = 0.0027 nM [10], inhibitor dissociation constant K_i_ = 0.005 nM [44], and K_i_ = 0.53 - 2 nM [45] respectively).

When major mutations (e.g. at positions 46, 50, 54, 84, and 90) were introduced, as shown for ATV_0, IDV_0, IDV_1, LPV_1, and NFV_1 (Tables 1, 2 and 3), the binding pocket volume increased. This agrees with the in vitro findings that combinations of these mutations known as “quadruple mutant” caused multi-drug resistance [46].

To further investigate the mechanism, we analyzed the effects of these mutations on the PI-binding site using SPACER [39]. We found that both the major and minor mutations influenced the PI-binding pockets. The quadruple mutant-containing complexes (ATV_0, IDV_0, LPV_1, and NFV_1) showed increased allosteric effects when compared to the native complexes (Tables 1, 2 and 3). In addition, some minor mutations at position 82, demonstrated a strong effect on the binding region (in NFV_3, NFV_4, SQV_3, or SQV_4 complexes) when mutated to hydrophilic residues.

To investigate the structural stability of the mutated proteases, we first checked for possible biases in side chain replacement by SCWRL4 [35]. As a control, the replacement protocol was applied on the native protease [PDB: 1ODW], where all the side chains were first removed and added back to the retained backbone. Our results of superimposed full structures of the original native and the reconstituted structures showed that SCWL4 was able to recover the side chains of the native structure (RMSd ~ 0 Å). Thereafter, SCWRL4 was used to model the mutated side chains for all the protease mutants. ENCoM [41] was used to estimate the free energy and vibrational entropy and we found that the mutations destabilized the protease structure even though they caused reductions of PI susceptibility (Tables 1, 2, 3 and 4). In addition, it was shown that mutations might have caused structural changes in some mutated protease structures when compared to the native protease (i.e. RMSd values in Tables 1, 2, 3 and 4). Yet these values are unlikely to reflect the structural effect caused by single mutations. Nonetheless, we have incorporated the RMSd factor into our integrated vector *C*_*v*_ to construct the correlation network. This was performed to include the structural contribution effect in the network, and also to avoid the possible biases toward allosteric communication and free energy.

**Fig. 1:**
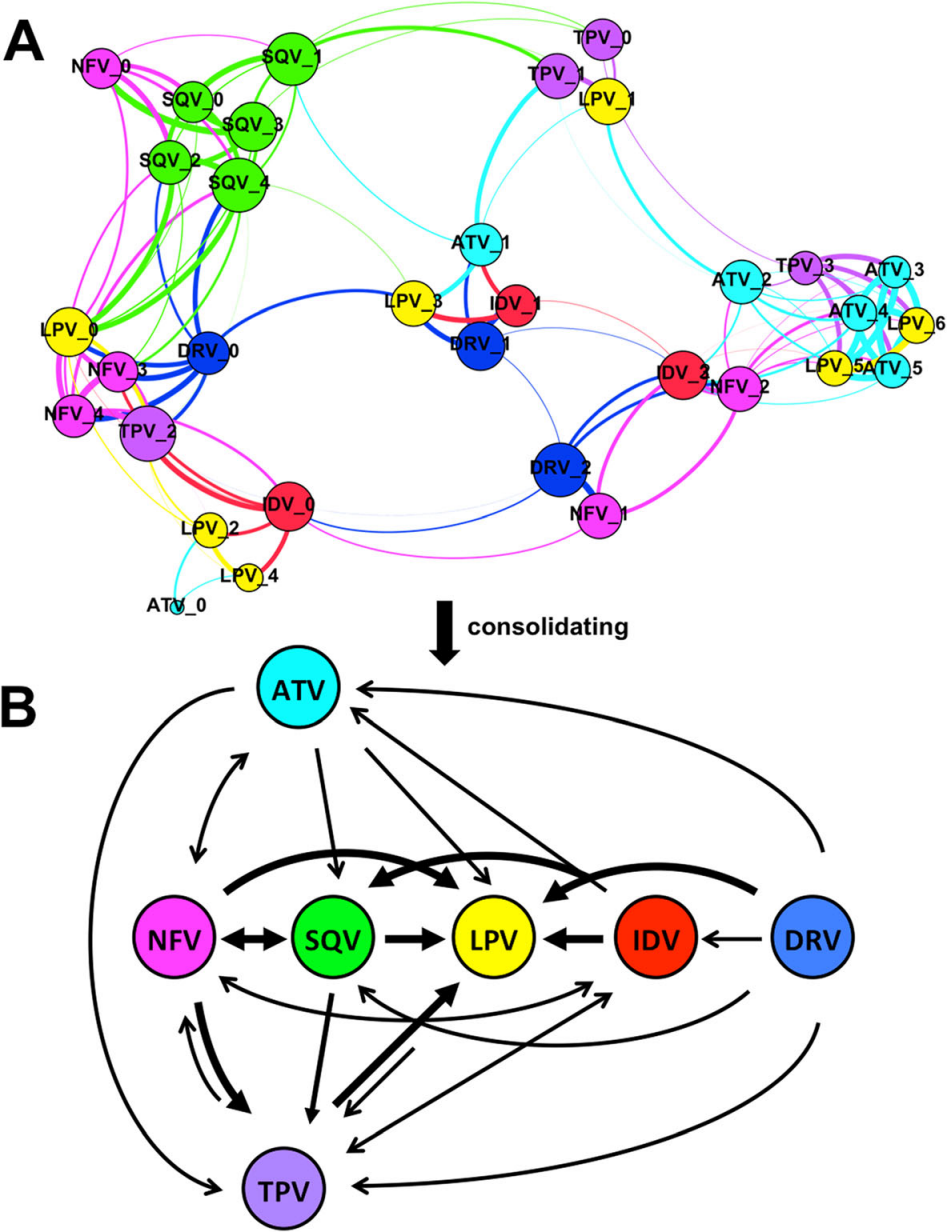
Structure-based correlation network of the mutant protease-PI complexes. **a** Thirty-three protease-PI mutant complexes (represented by nodes) are coloured according to seven PIs: Atazanavir (ATV, *cyan*), Darunavir (DRV, *blue*), Indinavir (IDV, *red*), Lopinavir (LPV, *yellow*), Nefinavir (NFV, *magenta*), Saquinavir (SQV, *green*), and Tipranavir (TPV, *purple*). The protease-PI complexes are numbered following indices: 0 (the PDB available complex structure that was used to initiate the reported mutations), 1 (mutated protease-PI complexes containing both major and minor mutations), the rest from 2 to 6 (mutated protease-PI complexes containing minor mutations). In this network, edges are coloured based on the source node, which are connected to other nodes; hence highlighting the link from one node to the others. The node sizes are varied based on different related closeness level of each node to other nodes. **b** A consolidated graph that shows predominant trends that the protease is most likely to resist, from one PI to another. The thicker the arrows are, the more likelihood that protease mutations extend the resistance to the corresponding PIs. The graph was generated using Gephi v0.82 [37]

Using the multiple structural constraints shown in Tables 1, 2, 3 and 4, we generated a structure-based correlation network of the mutant protease-PI complexes with respect to various PIs (Fig. 1). Figure 1 shows that mutations associated with the seven PIs are highly related where cross-resistance could develop easily against NFV, SQV, LPV, IDV, or DRV (Fig. 1b), with the arrows indicating the predominant directions of the correlations. The results suggest that LPV should be used as the first line of treatment to reduce the development of cross-resistance (since the arrows point from the other drugs to LPV and not vice versa, see Fig. 1b).

**Fig. 2:**
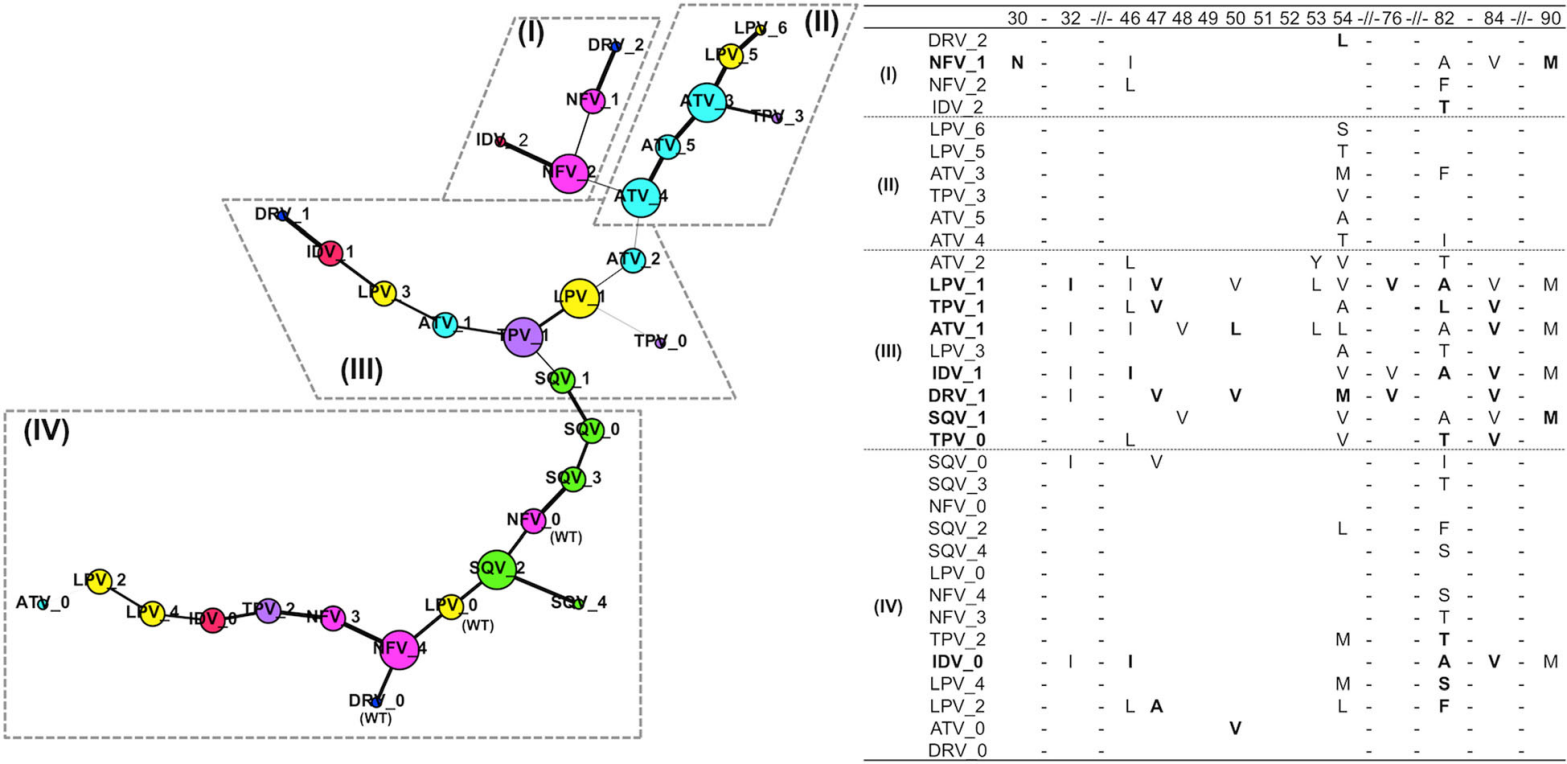
The drug resistance-related pathway that Protease might travel to resist from one PI to another. The minimum spanning tree or MST (*left*) shows the shortest path that leads to different PI resistance from a wild type protease (e.g. complex of protease-DRV at the bottom of the MST). Nodes are coloured and scaled the same as shown in Fig. 1 according to seven PIs: Atazanavir (ATV, *cyan*), Darunavir (DRV, *blue*), Indinavir (IDV, *red*), Lopinavir (LPV, *yellow*), Nefinavir (NFV, *magenta*), Saquinavir (SQV, *green*), and Tipranavir (TPV, *purple*). Edge thickness is weighted based on correlation between nodes. Highlighted on the right are the major (in *bold*) and minor mutations that could distinguish four close groups of mutant protease complexes (namely group I, II, III, and IV): PI-binding site region (position 30, 32), protease flap region (position 46–54), and other important major mutations (position 76, 82, 84, and 90). Protease-PI complexes containing major mutations are highlighted in *bold*. The graph was generated using Gephi v0.82 [37]

We next constructed a MST to extract the shortest path between the different PI resistant proteases from a wild type protease (i.e. complex of protease-DRV at the bottom of the MST). Our MST demonstrates a path (Fig. 2) for a wild type protease to develop resistance from one PI to another, serving as a guide for the structural relationships among different protease mutants to various PIs.

The MST was sectioned into four main groups: (I), (II), (III), and (IV) as shown in Fig. 2. The major group III (including most of major mutations-containing protease-PI complexes, i.e. DRV_1, IDV_1, ATV_1, TPV_1, LPV_1, and SQV_1) was connected to the other groups at ATV_2 (to group II) and at SQV_1 (to group IV). The other major resistant mutant NFV_1 however belonged to group I. As the connections between the mutant complexes were weighted based on their structural correlations (see Methods), the thicker edges demonstrated the high likelihood of cross-resistance to the next PI in the tree.

In the major group III, strong connections between protease mutants that resist DRV (i.e. DRV_1) and IDV (i.e. IDV_1) were observed. This connection was supported by clinical findings where the combinations of mutations V32I, I47A, and L76V, conferred high-level resistance to DRV and IDV [34]. The path thus further dissuades the use of DRV on patients with IDV resistant viruses.

Interestingly, the MST distinguished NFV_1 from the other major mutants. A distinct mutation D30N around the NFV-binding region might characterize the specificity of the NFV_1 complex (Fig. 2 and Table 3). However, this D30N mutation is rarely associated with NFV-resistance in some non-subtype B HIV [34], suggesting that NFV can be used as a first-line therapy to avoid the rapid development of resistance to other PIs.

Our structural and sequence analyses showed that mutations at the protease flap regions (position 46–54) and residue 90 (i.e. L90M) played a key role to distinguish group III from the other groups. In the major resistance protease mutants (i.e. group III), the flap region mutations were predominantly hydrophobic and accompanied by mutations at position 90 (e.g. L90M), whereas in the other minor resistance protease mutants, the flap regions were mostly hydrophilic with no mutations occurring at position 90 (Fig. 2). The physicochemical changes of the flap regions may have caused rigid or buried flaps, resulting in reduced PI susceptibility.

However, an exception to this trend was found for I50L in the mutant ATV_1 previously reported to improve susceptibility to IDV, SQV, LPV, or NFV [47–49]. In our MST, we further defined this relationship where ATV_1 was closer to IDV_1, SQV_1, and LPV_1 than to NFV_1, suggesting that NFV_1 resistance would develop slower when compared to the others. On the other hand, the other drug resistance mutations (IDV_1, SQV_1, LPV_1) would develop intervening intermediates (LPV_3 for IDV_1 and TPV_1 for the latter two).

For group IV, the path depicted that patients first treated with NFV (i.e. NFV_0 –wild type protease) may develop significant changes, e.g. in the flap region at position 54, and at the hotspot 82 in order to resist SQV. For patients treated with LPV (i.e. LPV_0 – wild type protease), there may be a series of resistance developed against NFV, SQV, and TPV by a single or a few minor mutations (Fig. 2, group IV). This suggests for the displacement of SQV instead of NFV in clinical use to avoid rendering LPV ineffective later.

### Detecting protease structure stability-affecting hotspots

Major mutations typically confer PI resistance but also cause viral loss of fitness. This loss is often in turn compensated by other minor mutations. In our findings, we showed that the major mutations destabilized protease structure via increased vibrational entropy (calculated by ENCoM [41]), and this vibrational entropy contributed significantly to the free energy of proteins [50]. To evaluate the reliability of the ENCoM findings, we used CUPSAT server [51] and found 63% (12/19) agreement in the calculation of the single mutations.

**Fig. 3:**
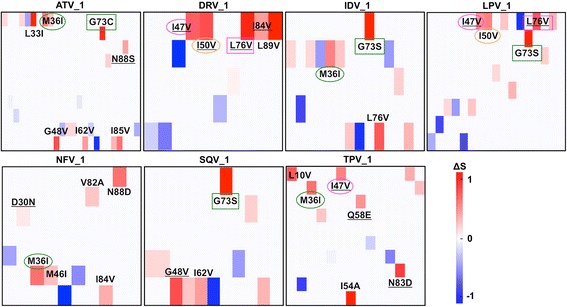
Mutation hotspots that cause protease structures to be unstable when resisting PIs. The hotspots were detected based on vibrational entropy (ΔS) using ENCoM [41] for seven major mutation-containing protease-PI complexes: Atazanavir (ATV_1), Darunavir (DRV_1), Indinavir (IDV_1), Lopinavir (LPV_1), Nefinavir (NFV_1), Saquinavir (SQV_1), and Tipranavir (TPV_1). The major mutations that reduced the PI susceptibility are underlined. Common hotspots among PIs are highlighted in coloured boxes and ovals. The size of the residue columns is reciprocal to the total number of mutations in each complex. Heat maps are generated by the ENCoM

As shown in Fig. 3, many minor and even major mutations caused protease structure to be unstable in the course of resistance development. Resistance to DRV, LPV, and TPV resulted in noticeable stability impact as they contain the destabilizing major mutations (Tables 1, 2, and 4). With 60% (6/10) mutations causing the instability, viral resistance to escape from the strong DRV binding [10] comes with significant structural stability compromise.

Of these mutations, residue substitutions at the flap regions I47V (in all PI resistance), I50V (in DRV and LPV), and the mutation L76V (in DRV and LPV) significantly increased the regional entropy. While similar protease-destabilizing effect by major mutations was observed in LPV and TPV resistance, the minor mutation G73S (LPV) or I54A (TPV) amplified this effect while compensating for the viral fitness loss.

## Conclusion

Based on the structural responses and viral fitness cost of the clinically reported mutations, we report a PI resistance-related pathway that HIV protease may undertake in PI cross-resistance. In this we found the structural rationale for the rapid development of cross- resistance amongst five of the seven clinically used PIs. There are some PIs that would be better used in clinical settings against naïve HIV infections or when resistance has already been developed towards another PI. On this, our findings suggest the use of LPV as the first line of PI in HAART, and depending on the emergence of PI-resistant mutations, certain drugs would be more useful in subsequent lines of treatment. The findings of this study thus provides a structural understanding that may be useful to guide the clinical use of PIs in HAART, aiding in drug selection to prolong the effectiveness of the given PI.

## Additional file

Additional file 1: Dynamic changes of pocket volumes of three wild type protease-PI complexes during 10 ns molecular dynamics simulation. The three wild type protease-PI complexes (DRV_0, LPV_0, and NFV_0) contain single residue substitutions S37N, L63P, and V3I respectively that contributed to shrink the PI-binding pocket as compared to the native protease [PDB:1ODW]. The fluctuation of pocket volume of the native protease is estimated based on the three PIs (DRV, LPV, and NFV), and is shown in gray shade (~1955 ± 213 Å^3^). The molecular dynamics simulations were performed in standard protocol for 2x5ns using AMBER14. (PDF 564 kb)

## Abbreviations

AIDS: Acquired immunodeficiency syndrome
ATV: Atazanavir
DRV: Darunavir
HAART: Highly active anti-retroviral therapy
HIV: Human immunodeficiency virus
IDV: Indinavir
LPV: Lopinavir
MST: Minimum spanning tree
NFV: Nefinavir
PIs: Protease inhibitors
SQV: Saquinavir
TPV: Tipranavir
