# Supplementary material for: Structural analyses of 2015-updated drug-resistant mutations in HIV-1 protease: an implication of protease inhibitor cross-resistance
Source: BMC Bioinformatics. 2016 Dec 22;17(Suppl 19):500. doi: 10.1186/s12859-016-1372-3 (PMC5259968; doi:10.1186/s12859-016-1372-3)
Supplement: Additional file 1: — Dynamic changes of pocket volumes of three wild type protease-PI complexes during 10 ns molecular dynamics simulation. The three wild type protease-PI complexes (DRV_0, LPV_0, and NFV_0) contain single residue substitutions S37N, L63P, and V3I respectively that contributed to shrink the PI-binding pocket as compared to the native protease [PDB:1ODW]. The fluctuation of pocket volume of the native protease is estimated based on the three PIs (DRV, LPV, and NFV), and is shown in gray shade (~1955 ± 213 Å3). The molecular dynamics simulations were performed in standard protocol for 2x5ns using AMBER14. (PDF 564 kb) [file 12859_2016_1372_MOESM1_ESM.pdf]

Additional File 1:

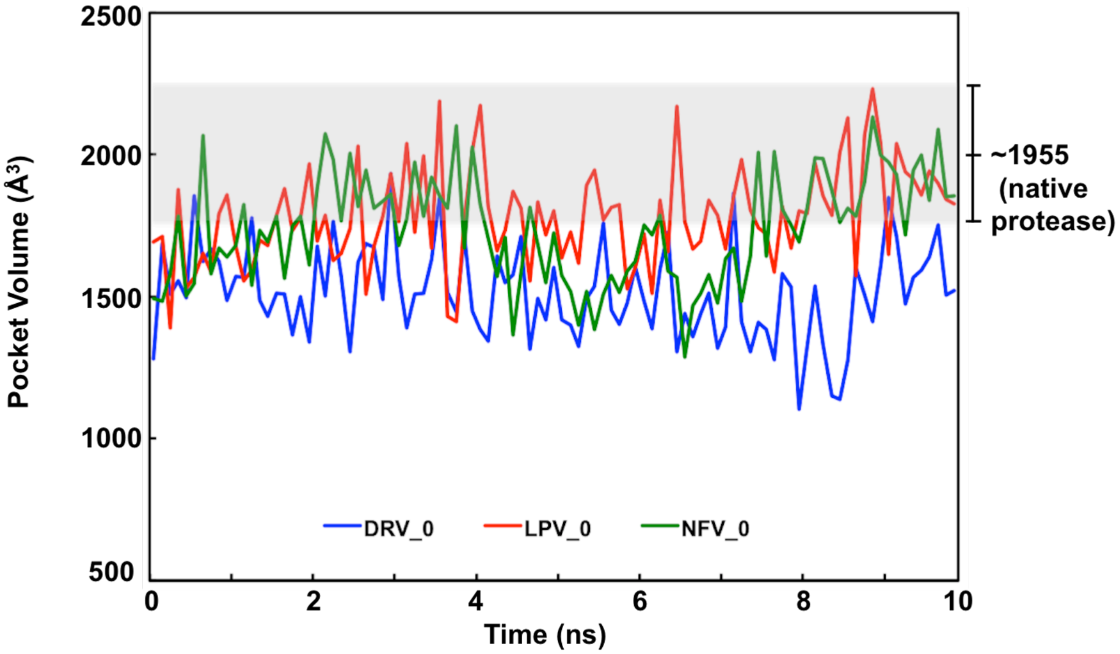

Figure S1: Dynamic changes of pocket volumes of three wild type protease-PI complexes during 10ns molecular dynamics simulation.

The three wild type protease-PI complexes (DRV_0, LPV_0, and NFV_0) contain single residue substitutions S37N, L63P, and V3I respectively that contributed to shrink the PI-binding pocket as compared to the native protease [PDB:1ODW]. The fluctuation of pocket volume of the native protease is estimated based on the three PIs (DRV, LPV, and NFV), and is shown in gray shade (~1955 ± 213 $Å^3$).

The molecular dynamics simulations were performed in standard protocol for 2x5ns using AMBER14 [1].

1. Case DA, Berryman JT, Betz RM, Cerutti DS, T.E. Cheatham I, Darden TA, Duke RE, Giese TJ, Gohlke H, Goetz AW *et al*: **AMBER 2015.** In. University of California, San Francisco; 2015.
